# Aβ Clearance, “hub” of Multiple Deficiencies Leading to Alzheimer Disease

**DOI:** 10.3389/fnagi.2015.00200

**Published:** 2015-10-20

**Authors:** Pasquale Gallina, Antonio Scollato, Renato Conti, Nicola Di Lorenzo, Berardino Porfirio

**Affiliations:** ^1^Department of Surgery and Translational Medicine, University of Florence, Florence, Italy; ^2^Neurosurgery Unit, Head and Neck Department, University Hospital “Ospedali Riuniti” of Trieste, Cattinara, Italy; ^3^Department of Clinical and Experimental Biomedical Sciences “Mario Serio”, University of Florence, Florence, Italy

**Keywords:** Alzheimer disease, Aβ clearance, aging, apolipoprotein E, neuroinflammation, Virchow–Robin spaces, neurovascular unit

The role of the amyloid cascade in the pathogenesis of Alzheimer’s disease (AD) is still the subject of passionate debates (Herrup, 2015; Musiek and Holtzman, 2015). According to quite a radical viewpoint (Herrup, 2015), the tendency to try to find a unifying etiopathogenetic pathway has so far hampered the understanding of such a complex disease. Thus, it would be better to reject the amyloid cascade hypothesis since it is neither necessary nor sufficient to drive the development and progression of AD. Herrup (2015) proposes, as food for thought, to relocate the amyloid cascade in a multifactorial context where it represents only one of a number of deficiencies contributing to degenerative escalation in the age-weakened brain (Herrup, 2015). From a more conservative perspective, the amyloid cascade is the necessary key initiator of a complex sequence of pathological changes, especially tau protein hyperphosphorylation, which mediates neurodegeneration (Musiek and Holtzman, 2015). However, owing to the lapse in time between the appearance of amyloid plaques and that of tau protein tangles, neuronal loss and dementia, as well as the absence of an obvious anatomical colocalization between the amyloidogenic process and neurodegeneration areas, the amyloid cascade hypothesis is not sufficient to explain AD pathology unless supported by a series of “wingmen” (Musiek and Holtzman, 2015).

In our view, the case is neither for rejecting the amyloid cascade hypothesis, which would be equivalent to throwing out the baby with the bath water, nor for invoking “wingmen” without which the amyloid cascade hypothesis would not hold water, because the foremost, necessary “wingman” is a core element within the amyloid hypothesis itself. Indeed, the disease process “…. is proposed to result from an imbalance between Aβ production and Aβ clearance” (Hardy and Selkoe, 2002). The imbalance between Aβ production and disposal mainly comes from overproduction in familial AD (Scheuner et al., 1996), while in sporadic cases it may be due to a reduced ability to clear (Mawuenyega et al., 2010). One way or another, the role of Aβ clearance in AD continues to be recognizably underscored (Bohm et al., 2015). There are several ways by which Aβ is cleared from the brain. They include proteolytic degradation in both intracellular and extracellular parenchymal compartments, either fluid-phase macropinocytosis or receptor-mediated cellular uptake by astrocytes and microglia followed by lysosomal degradation, and bulk flow from interstitial fluid (ISF) to blood and lymphatic systems (Bohm et al., 2015).

In light of the discovery of novel efflux routes, which dump waste molecules away from the interstitial space, we think it is currently possible to reconsider the role of Aβ clearance in AD pathogenesis on a much better informed basis than ever before. The existence of a system responsible for drainage of the ISF from the brain parenchyma into the CSF, the so-called glymphatic system (Iliff et al., 2012), its relationship with the newly found meningeal lymphatic vessels in connection with deep cervical lymph nodes (Louveau et al., 2015), and their anatomo-physiological interplay with the intracranial vascular system (Beggs, 2013), provide a new outlook of the hydrodynamics of cerebral fluxes. There is attention focused now on the emerging role of Virchow–Robin spaces (VRS) in the homeostasis of cerebral fluids in the CNS (Brinker et al., 2014). Neuroanatomically, the VRS refer to perivascular compartments surrounding small arteries when penetrating from the subarachnoid space into brain parenchyma and the small veins leaving these compartments (Ishikawa et al., 2015). Lining the VRS is continuous astrocyte endfeet with a high expression of aquaporin-4 (Aqp4) water channels allowing CSF flux to pick up exhausted material from the ISF and flush it out along paravenous drainage pathways (Iliff et al., 2012). Thus, VRS are the site where interstial and cerebrospinal fluids, vessels, and brain parenchymal components meet to constitute the neurovascular unit (Stanimirovic and Friedman, 2012). If Aqp4 localization is lost, or if CSF outflow is reduced as a consequence of either CSF flow obstruction or cerebral artery pulsatility inefficiency, or cerebrospinal venous insufficiency and lymphatic disorders (Brinker et al., 2014), local perivascular CSF recirculation may be impaired and, consequently, the VRS may dilate due to fluid retention (Weller et al., 2015). No wonder that it has recently been proposed to rename the dilated VRS as dilated interstitial spaces (Ishikawa et al., 2015). When VRS dilatation occurs, the neurovascular unit may be damaged through either direct compression or the resulting ischemic insult expanding outward on the surrounding parenchyma, and inward on blood vessels. A reduced CSF turnover results in accumulation and stagnation of byproducts at the level of the VRS. This can further hurt the neurovascular unit by inducing a proinflammatory milieu and by maintaining/worsening clearance damage in a self-feeding fashion. Note that CSF diversion, while favoring CSF clearance and/or reducing parenchymal compression, was beneficial in a small series of patients with normal pressure hydrocephalus-like symptoms associated with severe VRS dilatation (Scollato et al., 2015). The relevance of ISF–CSF flux disturbances in AD is suggested at the inner boundary of the system by experiments based on disruption of Aqp4 functions (Iliff et al., 2012), and at the olfactory archaic route of CSF outflow by the frequent observation of early olfactory dysfunction as a consequence of cribiform plate disruption (Ethell, 2014). Notably, a higher incidence of dilated VRS has been found in AD patients (Ramirez et al., 2015) also in association with Aβ deposition along the perivascular fluid drainage pathways of cortical and leptomeningeal arteries (Roher et al., 2003).

It has become increasingly apparent that, when looking at Aβ clearance impairment, special emphasis should be assigned to aging (Kress et al., 2014), inflammation (Heppner et al., 2015), and apolipoprotein E (ApoE) (Kanekiyo et al., 2014). Aging has been associated with a drastic decline in the efficiency of exchange between the subarchnoidal CSF and brain parenchyma due to both a reduction in penetrating arterial pulsatility and altered Aqp4 expression within astrocytes (Kress et al., 2014). Moreover, impairment of the “perivascular pump” driven by cerebral arterial pulsation (Hadaczek et al., 2006) may lead to CSF flux disturbance (Iliff et al., 2013), as seen in small vessel disease and normal pressure hydrocephalus, aging-related conditions that also share with AD the cardinal sign of cognitive decline and VRS enlargement (Wardlaw et al., 2013; Ishikawa et al., 2015). Another emerging issue in AD is neuroinflammation, which also helps explain clearance disturbances. Soluble Aβ oligomers are sensed by cell-surface receptors of the microglia, which is primed to cope with these and other misfolded proteins through glial-induced proteolitic degradation and phagocytosis (Heppner et al., 2015). Inflammation-related changes in the neurovascular unit eventually amplify neurodegeneration by self-sustaining amyloidogenic over-production and decreased brain clearance, even through derangement of astrocytic Aqp4 expression and distribution (Heppner et al., 2015). Without a doubt, the *APOE* genotype is the best-known factor with a sizable effect on AD in terms of occurrence risk and of onset age (Farrer et al., 1997). It has been shown that ApoE interacts with Aβ along all its clearance pathways (Kanekiyo et al., 2014) and that the presence of the ApoE4 isoform may adversely affect Aβ metabolism. In particular, oligomeric Aβ levels in the CSF are increased in AD patients compared to non-AD and are greater in *APOE4/4* compared to *APOE3/3* AD patients (Tai et al., 2013). Furthermore, there seems to be an inverse relationship between the levels of soluble ApoE/Aβ complex and oligomeric Aβ, suggesting that the complex plays a significant role in modulating oligomeric Aβ levels, either by affecting Aβ clearance or Aβ aggregation, or both. The lower levels and instability of ApoE4/Aβ complex compared to ApoE3/Aβ, possibly owing to the hypolipidated status of the ApoE4 isoform, suggest a possible mechanism for the ApoE4-induced risk for AD.

That is to say, the three main elements involved in AD all contribute to the damage of the cerebral hydrodynamic system at one or more levels. In addition to these elements acting either additively or synergically on AD, there certainly are several others, which are strongly overlapping and integrated. Genome-wide association studies have identified a handful of common variants in genes involved in lipid metabolism, immunity, inflammation, and endocytosis (Calero et al., 2015). However, they probably explain <25% of the genetic variance. Future AD genetics research with novel technologies may possibly identify rare, high penetrant variation that may account, at least in part, for the remaining genetic variance in AD. It is also possible that gene–gene interactions, somatic mutations, and epigenetics will eventually account for much of the unexplained genetic and phenotypic variance in AD. Altogether, these analyses highlight the value of defining pathways and networks of gene–environment interactions rather than the contribution of individual genes (Calero et al., 2015). It is also well known that environmental factors such as trauma and detrimental life styles may have a role (Fotuhi et al., 2009). Paradigmatically, detrimental life styles accelerate age-related, ApoE4-dependent, inflammation-mediated cardiovascular and cerebrovascular damage, while feeding the risk of developing AD (Fotuhi et al., 2009), possibly through the derangement of one or more Aβ clearance pathways. Sleep and circadian rhythm disturbances are common in aging and AD patients (Fotuhi et al., 2009). Sleep deprivation leads to glymphatic Aβ clearance impairment in the mouse, possibly linked to a reduction of the interstitial space width, insofar as it would increase resistance to convective fluid movement (Xie et al., 2013). On the other hand, amyloid plaque formation may cause sleep disruption, leading to a feedback loop of further Aβ deposition either directly (Ju et al., 2014) or through neuroinflammation (Wisor et al., 2011). Another important aspect of a healthy life style is exercise, which has been shown to improve Aβ clearance in mouse models of AD (Nichol et al., 2008; Lin et al., 2015). Moreover, physical activity has been found to be associated with delayed Aβ deposition in preclinical AD (Okonkwo et al., 2014), while cognitively normal sedentary *APOE4* carriers may be at augmented risk for cerebral amyloid deposition (Head et al., 2012). As far as nutrition is concerned, saturated fatty acid intake may adversely affect cognition (Morris et al., 2004), possibly through mechanisms involving ApoE (Laitinen et al., 2006) and/or conveying inflammation (Timmermans et al., 2014). Insufficient antioxidant dietary supply can elicit a chronic inflammatory response, where the cytotoxic properties of soluble Aβ oligomers are mediated via a reactive oxygen species pathway (Dumery et al., 2001; Giordano et al., 2014). Moreover, exposure to a fat-rich diet during gestation and lactation has far-reaching consequences over the entire lifespan in adult mice because of impaired perivascular Aβ clearance from the brain (Hawkes et al., 2015). The elements we associated to clearance impairment in this contention are among those deficiencies listed by Herrup (2015) and identified as “wingmen” in the model discussed by Musiek and Holtzman (2015). Additional deficiencies should be included in our hydrodynamic reappraisal. Even though it is known that traumatic brain injury is associated with AD, only recently have experiments demonstrated that tau pathology and onset of neurodegeneration are dependent on an impaired glymphatic system (Iliff et al., 2013). As a novel category of deficiencies to be taken into account, we hereby introduce CSF outflow disorders. It is now emerging that chronic cerebrospinal venous insufficiency plays a role in the dynamics of white matter hyperintensities in AD patients (Chung et al., 2014), since it may lead to CSF outflow reduction (Beggs, 2013). Notably, these imaging features can have VRS dilatation as pathological substrate and represent a biomarker that AD has in common with small vessels disease, cerebral amyloid angiopathy and normal pressure hydrocephalus. Disorders of the lymphatic system of the brain, as occur in aging and in association with *APOE4*, may result in clearance impairment and AD development (Pappolla et al., 2014; Weller et al., 2015).

Ultimately, the hydrodynamic refocusing on AD is consistent on the one hand with the mainstream hierarchical model of the amyloid cascade (Hardy and Selkoe, 2002) in a scheme where multiple deficiencies lead to Aβ clearance impairment, the “hub” which drives downstream processes (Figure 1). On the other hand, it seems to solve the spatial-temporal issue raised by Herrup (2015) and by Musiek and Holtzman (2015). While the plaque burden may not be specifically correlated with cognitive dysfunction in AD patients, there is consensus that soluble Aβ42 and oligomeric Aβ represent major proximal, neurotoxic species in AD (Hardy and Selkoe, 2002). The ISF–CSF flow allows the movement of a variety of cytotoxic oligomeric Aβ species in the brain. This makes tau triggering by Aβ possible in various areas apart from where the largest deposits occur. At the same time, with aging, Aβ aggregates are the first to be difficult to be rid of compared to soluble types, which could explain why they are visible many years prior to the onset of other pathogenetic events. A 20- to 30-year interval between amyloid positivity and dementia has recently been confirmed in large studies (Jansen et al., 2015). In our refocusing, this window offers a wide margin for tailored preventative intervention, spanning from enhancement of ISF–CSF clearance by promoting healthy lifestyles to correction of hydrodynamic regulatory system disturbances.

**Figure 1:**
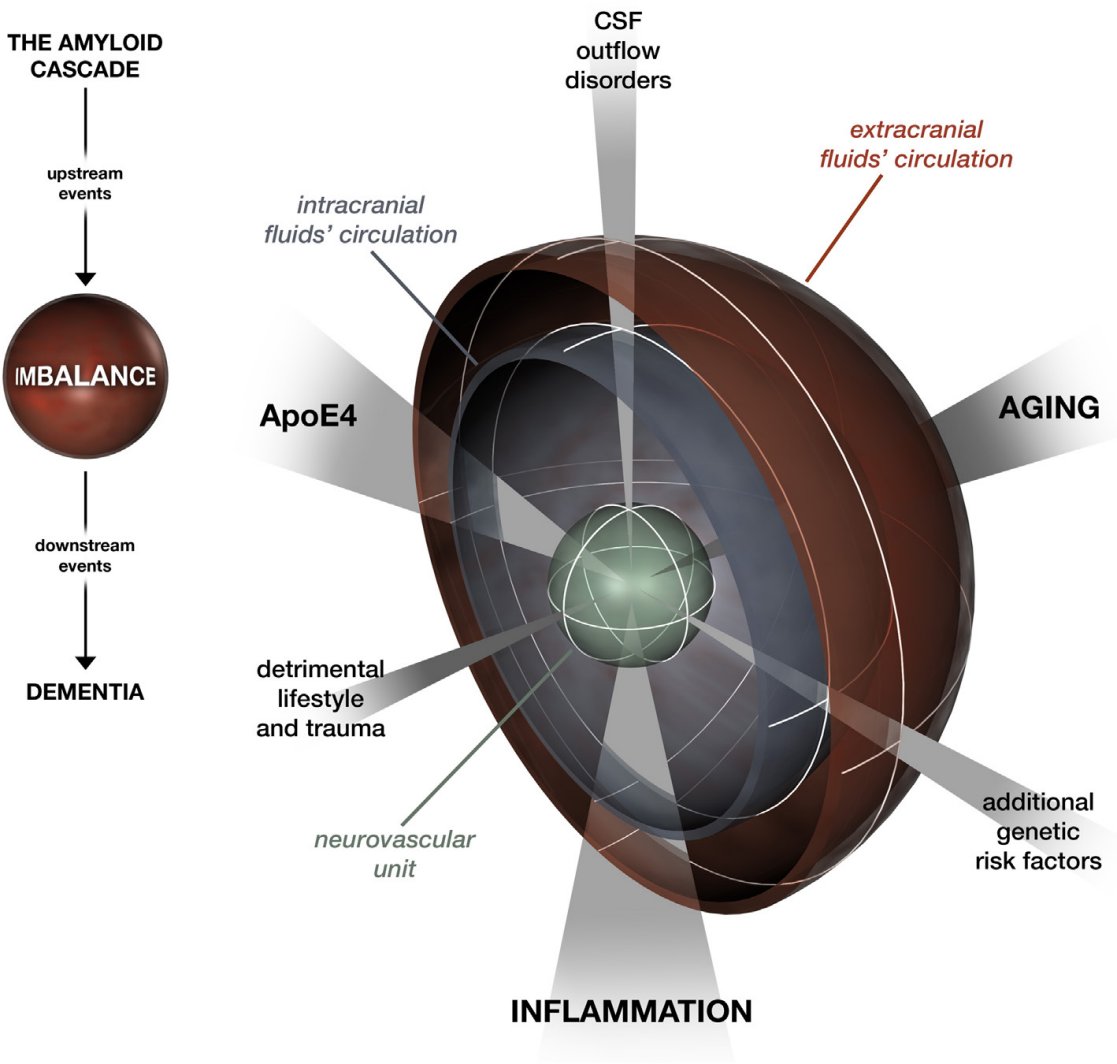
**A look at the amyloid cascade hypothesis focusing on the imbalance between A**β** production and clearance**. Aging, inflammation, and ApoE4 affect Aβ clearance by networking at the level of the neurovascular unit and the intra- and extracranial fluid circulation systems. Likewise, modifiers that feed the imbalance are categorized under three domains: detrimental lifestyle and trauma, additional genetic risk factors, and CSF outflow disorders.

## Conflict of Interest Statement

The authors declare that the research was conducted in the absence of any commercial or financial relationships that could be construed as a potential conflict of interest.
